# Voronoi distance based prospective space-time scans for point data sets: a dengue fever cluster analysis in a southeast Brazilian town

**DOI:** 10.1186/1476-072X-10-29

**Published:** 2011-04-23

**Authors:** Luiz H Duczmal, Gladston JP Moreira, Denise Burgarelli, Ricardo HC Takahashi, Flávia CO Magalhães, Emerson C Bodevan

**Affiliations:** 1 Department of Statistics, Universidade Federal de Minas Gerais, Campus Pampulha, Belo Horizonte/MG, Brazil; 2 Department of Mathematics, Universidade Federal de Ouro Preto, Ouro Preto/MG, Brazil; 3 Department of Mathematics, Universidade Federal de Minas Gerais, Campus Pampulha, Belo Horizonte/MG, Brazil; 4 Medical Doctor, Prefeitura de Belo Horizonte/MG, Brazil; 5 Department of Mathematics and Statistics, Universidade Federal dos Vales do Jequitinhonha e Mucuri, Diamantina/MG, Brazil; 6 Department of Electrical Engineering, Universidade Federal de Minas Gerais, Campus Pampulha, Belo Horizonte/MG, Brazil

## Abstract

**Background:**

The Prospective Space-Time scan statistic (PST) is widely used for the evaluation of space-time clusters of point event data. Usually a window of cylindrical shape is employed, with a circular or elliptical base in the space domain. Recently, the concept of Minimum Spanning Tree (MST) was applied to specify the set of potential clusters, through the Density-Equalizing Euclidean MST (DEEMST) method, for the detection of arbitrarily shaped clusters. The original map is cartogram transformed, such that the control points are spread uniformly. That method is quite effective, but the cartogram construction is computationally expensive and complicated.

**Results:**

A fast method for the detection and inference of point data set space-time disease clusters is presented, the Voronoi Based Scan (VBScan). A Voronoi diagram is built for points representing population individuals (cases and controls). The number of Voronoi cells boundaries intercepted by the line segment joining two cases points defines the Voronoi distance between those points. That distance is used to approximate the density of the heterogeneous population and build the Voronoi distance MST linking the cases. The successive removal of edges from the Voronoi distance MST generates sub-trees which are the potential space-time clusters. Finally, those clusters are evaluated through the scan statistic. Monte Carlo replications of the original data are used to evaluate the significance of the clusters. An application for dengue fever in a small Brazilian city is presented.

**Conclusions:**

The ability to promptly detect space-time clusters of disease outbreaks, when the number of individuals is large, was shown to be feasible, due to the reduced computational load of VBScan. Instead of changing the map, VBScan modifies the metric used to define the distance between cases, without requiring the cartogram construction. Numerical simulations showed that VBScan has higher power of detection, sensitivity and positive predicted value than the Elliptic PST. Furthermore, as VBScan also incorporates topological information from the point neighborhood structure, in addition to the usual geometric information, it is more robust than purely geometric methods such as the elliptic scan. Those advantages were illustrated in a real setting for dengue fever space-time clusters.

## Background

### Introduction

Algorithms for the detection and inference of clusters are useful tools in etiological studies [1] and in the early warning of infectious disease outbreaks [2-6]. A spatial cluster is defined as a localized portion of the domain containing a higher than average proportion of cases over controls, whose appearance is unlikely under the assumption that cases are randomly distributed in the population. *Space-time clusters *are defined as unexpected concentrations of disease cases in a time series sequence of geographical maps, and could potentially indicate an outbreak or epidemic, due to environmental or biological causes.

The spatial scan statistic [7] constitutes the main technique used for cluster detection, being employed, for instance, by the software packages SaTScan [8] and ClusterSeer [9] to detect static circularly shaped disease clusters [10]. Recently, several attempts have been developed in order to relax the assumption of cluster circular shape. Sahajpal et al. [11] used a genetic algorithm to find clusters shaped as intersections of circles of different sizes and centers. The SaTScan approach has been extended to the case of elliptic shaped clusters [4], in this way allowing the detection of elongated clusters. Conley et al. [12] proposed a genetic algorithm to explore a configuration space of multiple agglomerations of ellipses in point data set maps, implemented in the software PROCLUDE. Other methods have also been proposed to detect connected clusters of irregular shape [13-19]. A key point for the construction of such methods for detection of irregularly shaped clusters is that, as the geometrical shape receives more degrees of freedom, some correction should be employed in order to compensate the increased flexibility, so avoiding the increase of false-positive errors [16,20]. This fact has been recognized since the early study of elliptically shaped clusters [4]. Yiannakoulias et al. proposed a topological penalty [21]. These corrections were also treated in a multi-objective framework [17,22,23].

Neill's Fast Subset Scan [19] presented a significant advance in spatial methods for aggregated area maps, finding exactly the optimal irregularly spatial clusters in linear computing time. The clusters found may sometimes be disconnected, but this is not a serious disadvantage, provided that there is not a huge gap between its areas. A way to control the presence of those potential gaps is to limit the number of component areas of the cluster, e.g. allowing only clusters which are subsets of a circular zone of moderate maximum size.

These developments related to flexible cluster shapes have been mostly performed for the static case only. For the space-time case, the Prospective Space-Time Scan [24] considers all cylindrical clusters in the space-time domain as cluster candidates. A version of Space-Time Scan has been developed too for the case of the elliptical scan, also considering cylindrical clusters stated as projections of the ellipses along the time dimension [4]. The main motivation of this paper is the observation that, although the elliptical spatial shape endows some flexibility to the scan procedure, allowing a high detection power in space coordinates, the cylinder shape assumed in order to extend such a spatial shape to time coordinates is too restrictive, leading to inaccuracies in space-time cluster detection. This issue has been dealt in some references [25-27]. See [28] for a review of space-time cluster detection software.

Our proposed methodology builds different graphs for each considered time interval. In this way, the flexibility that is necessary for dealing with the variation of the disease spread along the time dimension is obtained in a direct way. In the next sections, a review of the spatial scan statistic introduced by Kulldorff and the prospective space-time scan is presented. Then, we introduce the novel space-time cluster detection algorithm for point data sets, evaluating it through numerical simulations. Finally, we apply the proposed method to find space-time clusters of dengue fever at individual level in Lassance City, located in the state of Minas Gerais, Brazil.

### The Spatial Scan Statistic

In this section we review the classical spatial scan statistic introduced by [7]. A point data set represents the location of individuals in a population of size *N *, classified either as controls or disease cases with *C *total cases. Under the null hypothesis there are no clusters and *μ_z _*is the expected number of disease cases in the window *Z*. Under the assumption of Poisson distribution, the logarithm of the likelihood ratio is(1)

where *c_z _*is the number of observed cases and 1(.) is the indicator function. This statistic is maximized over all the windows (potential clusters), identifying the zone that constitutes the most likely cluster.

The statistical significance of the most likely cluster of observed cases is computed through a Monte Carlo simulation, according to [29]. Under null hypothesis, simulated cases are distributed over the study region and the scan statistic is computed for the most likely cluster. This procedure is repeated thousands of times, and the distribution of the obtained values is compared with the LLR of the most likely cluster of observed cases, producing its p-value.

### Prospective Space-Time Scan

The Prospective Space-Time Scan [24] considers all cylindrical clusters in the space-time domain. All the possible circular windows in the space domain are taken as the bases of the cylinders to be considered. The study period is given by the time interval [*Y*_1_, *Y*_2_]. The likelihood for the observed data set is obtained as the maximum over all cylinders in the time interval [*s*, *t*] reaching the end of the study period, with *Y*_1 _≤ *s *≤ *t *= *Y*_2_. For the random data sets generated under null hypothesis, the likelihood is maximized over all cylinders for which *Y*_1 _≤ *s *≤ *t *≤ *Y*_2 _and *Y_m _*≤ *t*, where *Y_m _*is the time instant in which the time periodic surveillance began, in order to adjust for the multiple analysis. See [24] for details. SaTScan software implements the Prospective Space-Time Scan for both area and point data sets. In order to establish some comparisons for the evaluation of the proposed method, in this paper we have implemented a version of the Prospective Space-Time Scan for point data sets using elliptic cylinders instead of circular zones [4].

## Methods

The idea of employing a Minimum Spanning Tree (MST) in order to characterize clusters has been already studied in [30], in the context of area data sets. For dealing with point data sets, the application of the scan statistics requires a proper definition of disease case density related to each data point. As, clearly, a single sphere radius was not suitable for estimating the population density in all regions, due to the heterogeneity in the geographical distribution of population, a correction procedure was necessary. The procedure proposed by Wieland et al. [31] performed a non-linear cartogram transformation of the map, leading to a new map with an approximately homogeneous control population distribution. It should be noticed that this procedure is highly computing intensive.

In this paper, a much simpler procedure for the estimation of disease density is proposed. The general idea is: a Voronoi diagram is depicted, defining regions associated to each individual point in the map (both for disease and non-disease cases). A new distance, called Voronoi distance, between two points, is defined as the number of Voronoi cell boundaries that must be crossed in order to establish a path between those points. A ball of radius *R *in this distance, centered in the point *A*, would consist of the set of points which can be reached from *A *with up to *R *Voronoi cells crossings. Therefore, the Voronoi distance can be used in order to define a variable metric of the original coordinates that exactly performs the correction that transforms a non-homogeneous population density map into a homogeneous one. The computation of the Voronoi distance and all associated entities can be performed with efficient polynomial algorithms. Using the Voronoi MST, the computation of disease clusters in a fixed time coordinate can be performed very fast. In order to deal with space-time clusters, a simple procedure that connects the graphs of different time instants by the common nodes is employed. The program, written in Dev *C *language, is available from the corresponding author.

### Setting the Voronoi-Based Distance

In order to characterize point data set clusters the Voronoi distance is defined now. The population at risk consists of *N *individuals in the space domain, divided into *n *disease *cases *and *N *- *n controls*. Consider the set *P *= {(*x_i_*, *y_i_*): *i *= 1, ..., *N *} ⊂ ℝ^2^, indicating the geographic location of the cases and controls. For *i *= 1, ..., *N *the *Voronoi cell v*(*i*) consists of those points in ℝ^2 ^which are closer to (*x_i_*, *y_i_*) than to any other point in *P*. The *Voronoi diagram *is formed by the collection of cells *v*(*i*), *i *= 1, ..., *N*.

Let *v_ij _*be the number of Voronoi cells intercepted by the line segment joining the points (*x_i_*, *y_i_*) and (*x_j _, y_j_*) (including the cells containing the points *i *and *j*). In this work we define the *Voronoi distance *between points *i *and *j *as *δ*(*i*, *j*) = *v_ij _*- 1. When the points *i *and *j *occupy neighboring Voronoi cells, *δ*(*i*, *j*) = 1.

A geometric routine is used to compute the number of intersections of the segment linking two cases *i *and *j *with the edges of the Voronoi cells. If that segment intercepts tangentially a Voronoi cell, a potential problem may occur in the computation of *δ*(*i*, *j*). However, this problem occurs only rarely, supposing that the point coordinates follow a random pattern.

#### Set of possible clusters in space coordinates

Let *D *be a point data set. As an attempt to identify subsets of such a set that are likely to constitute a cluster, the following heuristic is employed here: A nonempty subset *S *of *D *forms a candidate cluster if the smallest distance separating the sets *S *and *D *- *S *is greater than the maximum internal distance of *S*, where *D *- *S *is the subset of *D *removing all points of *S*. Hence, the potential cluster is a connected graph with tree structure, linking the disease cases in the space domain. Our algorithm builds a set of sub-trees of the minimum spanning tree of the complete graph of cases, defining a small set of potential space clusters.

Formally, let *D *= {*c_i_*} be the subset representing the disease cases where each *c_i _*= (*x_i_*, *y_i_*) indicates its geographic location. We define a weighted complete graph *G*(*D*) = (*V*, *E*) with vertex set *V *= {*c_i _*: *c_i _*∈ *D*} and edge set *E *= {(*c_i_, c_j_*): *c_i_, c_j _*∈ *D*, *i *≠*j*}. Each edge (*c_i_, c_j_*) ∈ *E *has weight defined by the *Voronoi distance δ*(*i*, *j*). A minimum spanning tree MST of a weighted complete graph *G*(*D*) can be defined as a minimal set of edges of *G*(*D*) that connect all vertices with minimum total distance. The Voronoi Minimum Spanning Tree (VMST) of the weighted graph *G*(*D*) defined above is a spanning tree with the minimum total Voronoi distance. A set of discrete values characterizes the Voronoi distance. This would cause the emergence of multiple solutions very often. This effect is eliminated by ordering the edges with identical Voronoi distances according to the Euclidean distance. This procedure ensures the following lemma, which is an extension of the result proposed by [31]:

**Lemma 1 ***Assume that the Euclidean distance between any two points belonging to the set P is different from any other distance between two points of the same set. Then the set of potential clusters are in one-to-one correspondence with connected components among all graphs T_w_, with T_w _defined as the graph derived from VMST by deleting all edges having weight greater than w*.

*Proof: *Define the order of descending weights *w *to the edges of VMST untied by Euclidean distance as discussed above. Hence, the proof follows the same way as performed in [31], replacing the Euclidean distance by Voronoi distance.

The set of potential clusters may be quickly found from a VMST by using a greedy edge deletion procedure, improving and simplifying the strategy employed by the Density-Equalizing Euclidean MST method [31]. The procedure is: After constructing the Voronoi MST of the set of case locations *D*, we iteratively remove the largest remaining edge, giving rise to two additional cluster candidates in each iteration. For a map with *n *cases, we obtain 2*n *- 1 cluster candidates, including *n *unitary clusters.

**Figure 1:**
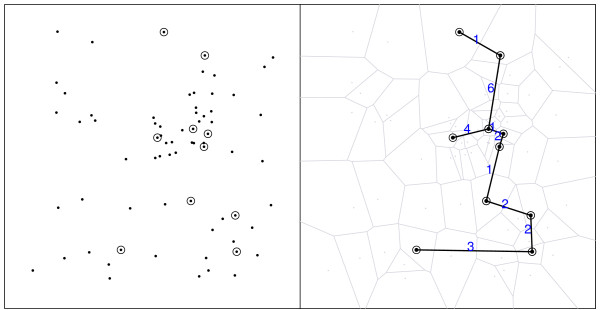
**Left: spatial distribution of the 10 observed cases (circles) and 70 non-cases (dots)**. Right: corresponding Voronoi minimum spanning tree.

**Figure 2:**
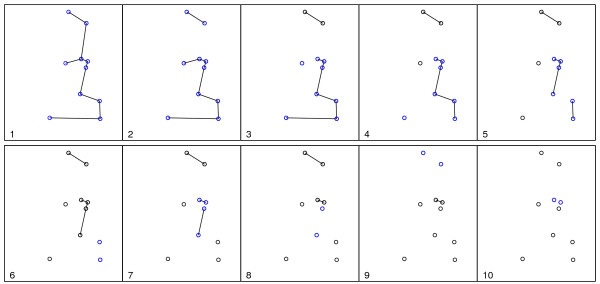
**Visualization of the greedy edge deletion procedure, in successive steps numbered from 1 to 10**. Sub-graphs linking blue circles represent the new cluster candidates that appear in each iteration, and sub-graphs linking black circles represent cluster candidates that have already appeared in former steps.

Figure 1 shows the spatial distribution of 70 coordinates, with 10 observed cases (circles) and 60 non-cases (dots) in an artificial data set and the associated Voronoi minimum spanning tree. Figure 2 shows a simple visualization of the greedy edge deletion procedure for the example above. The successive steps of edge deletion are represented, with the new cluster candidates shown in each iteration.

Given a case with geographic location *c_i _*= (*x_i_*, *y_i_*), consider the circle  centered in the point (*x_i_*, *y_i_*), with radius *r*. If the local density around the point (*x_i_*, *y_i_*) is given by *s *individuals per unit area, then the expected number of individuals inside the circle  is computed as *sπr*^2^. When the radius *r *is expressed locally in units of the Voronoi distance as *R*, then the expected number of individuals inside  is simply *πR*^2^. Thus the Voronoi distance definition contains the necessary information to compute approximately the local density function of the heterogeneous population, for a suitable choice of neighbors of each individual case.

**Proposition 1 ***Consider a case dataset D and its corresponding VMST, denoted by *. Let T *_S _be a connected subgraph of **whose nodes constitute the set S, and denote by f *(*x*) *the local population density in x. For each case c_i_***∈***S *let *ω_i _be equal to the minimum weight of the edges that are incident to c_i _in **and . The local population of S can be approximated by *.

This defines a "region of influence" of the cluster *S *through the composition of the regions of influence of each case, which are defined as circular regions, with radii *ω_i_*/2 chosen as large as possible, such that there is no interference between neighboring circles in the Voronoi MST.

We further note that this definition is robust, in the following sense. Consider two situations: first, a case dataset *D *spread evenly in a map of control points, and second, a case dataset *D*' with the same number of points and overall shape as *D *but geographically smaller, inserted in the same map of control points. It is easy to see that the regions of influence of the clusters associated to *D *is larger than the corresponding regions of influence associated with *D*', as we could expect.

We shall use this information to estimate the number of control individuals under the "region of influence" of each case individual, which in turn will allow the use of the scan statistic and also define a corresponding cluster finding algorithm employing a minimum spanning tree.

### Voronoi Space-Time Scan

In order to deal with space-time clusters, a simple procedure that connects the cases of different time instants for each time interval is employed. On what follows, we specify a parameter *τ *to indicate the maximum allowed temporal gap within the candidate cluster.

Let *P_T _*be the set of the geographic coordinates of the *N *- *n *controls and the *n_T _*disease cases present in the interval time window given by *T *= [*s*, *t*], where *s *is the initial time and *t *the final time of the interval *T*. The Voronoi diagram of *P_T _*and the corresponding Voronoi distance is defined similarly to the former procedure, in space coordinates only. For the space-time domain, let *t_i _*be the onset time of the disease for the *i*-th case, *i *= 1, ..., *n_T _*. Then, establish connections linking only cases whose temporal distance is limited by *τ*.

Formally, let be the set of cases observed in the interval *T *= [*s*, *t*], where *s *≤ *t_i _*≤ *t *and (*x_i_*, *y_i_*) indicates the geographic location for the case, *i *= 1, ..., *n_T _*. In this way, two observed cases will be connected if the temporal distance is such that |*t_i _*- *t_j_*| ≤ *τ*. We define a weighted complete graph *G^τ ^*(*D^T^*) = (*V^T ^*, *E^τ^*)

with vertex set

and edge set

The weights are the usual Voronoi distances between points (*x_i_*, *y_i_*) and (*x_j _*, *y_j_*).

The procedure is repeated for every time interval *T *= [*s*, *t*] such that *Y*_1 _≤ *s *≤ *t *= *Y*_2_, as seen in the Prospective Space-Time Scan section, building a different Voronoi based MST for each time interval *T*.

When using the parameter value *τ *= 1, the produced clusters of cases have no time gaps. Larger values of the parameter *τ*, otherwise, may produce clusters with cases separated by more than one unit of time, which could be undesirable in some circumstances. In the applications of the next section, we consider several possible values for *τ*.

## Results and Discussion

The Voronoi Based scans are compared through numerical simulations to the elliptic scan statistic. A data set of confirmed cases of Dengue fever in a small Brazilian city is presented. We apply the Voronoi Based Scan for the detection of Dengue fever clusters in space-time coordinates.

### Numerical Tests

In this section we present a set of numerical results. The Voronoi Based Scan (VBScan) was compared numerically with the elliptic version of the popular prospective space-time scan [24], according to power of detection, sensitivity and positive predictive value. Let {*X*_1_, *X*_2_, ..., *X_n_*} be random variables that denote the spatial coordinates of *n *cases observed in the data set. The sensitivity and positive predicted value (PPV) are defined as

A relative risk equal to 1.0 was set for every control outside the real cluster, and greater than 1.0 and identical in each control within the cluster. The relative risks for each cluster are defined such that if the exact location of the real cluster was known in advance, the power to detect it would be 0.999 [32].

In the first set of simulations, we evaluated only the spatial structure of the proposed algorithm.

#### A verification for purely spatial clusters

**Figure 3:**
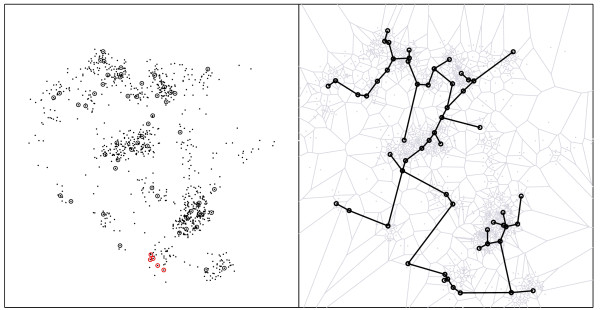
**Left: Spatial distribution of the observed cases (circles) and controls (dots) in Lancashire-UK, and the most likely cluster (red circles)**. Right: associated Voronoi minimum spanning tree.

The Voronoi based method, in its purely spatial setting, is applied for the well known data set of residential locations of larynx and lung cancer cases of the Chorley-Ribble area in Lancashire-UK, from 1973 to 1984. The 917 lung cancer cases are used as controls for the 57 larynx cancer cases (see http://cran.r-project.org/web/packages/splancs/splancs.pdf - pag. 55). In Figure 3 the spatial distribution of the observed cases (circles) and controls (dots) is shown on the left, and the Voronoi minimum spanning tree is shown on the right, with the Voronoi cells in the background. The elliptic spatial scan is also run as comparison. The p-values associated to the two scans are computed based on 9, 999 Monte-Carlo simulations under the null hypothesis. The most likely clusters found in both runs are identical, consisting of the five cases (red circles) of Figure 3. Table 1 shows the likelihood values, number of cases, p-values and running times for both scans. The set of possible elliptic clusters forms a more restrictive space of configurations than the set of of irregularly shaped clusters; not surprisingly, the elliptic scan p-value is smaller than the VBScan p-value, because the five cases in the most likely cluster fit very well inside an elongated ellipse.

**Table 1:** Comparisons spatial clusters detection of the cancer in Lancashire, match values to elliptic scan and VBScan methods.

Method	LLR	cases	p-value	CPU-Time(sec.)
Elliptic Scan	14.4049	5	0.0089	896
VBScan	10.8357	5	0.0470	449.5

#### Analysis of the Voronoi based space-time scan

We used artificial datasets with total population at risk of 1, 000 individuals, including 100 cases and 900 controls. The instances were simulated with a square space region [0, 1] × [0, 1] and a ten days time interval [1,10]. Space-time clusters with different shapes were considered. Numerical simulations were conducted using an artificial map constructed with the spatial locations of the individuals of the population at risk following an uniform point process, and the time of occurrence of the events following a discrete uniform distribution.

**Figure 4:**
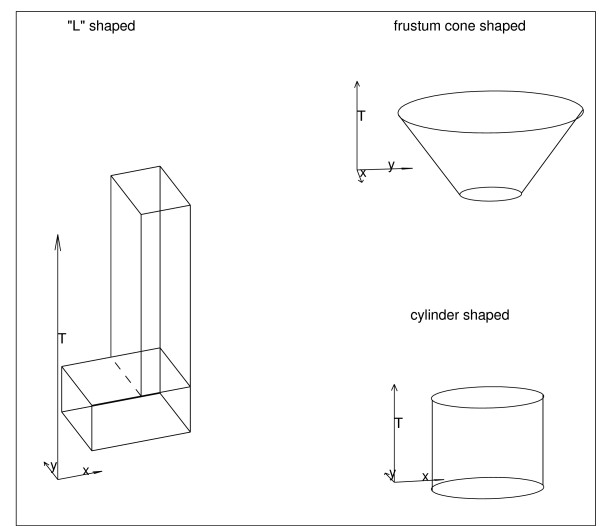
**Three alternative artificial space-time clusters**.

The Voronoi based method was compared to the prospective elliptic space-time scan statistic. Three alternative models of space-time clusters with different shapes were simulated. The three space-time cluster zones, as shown in Figure 4, aggregate spatial areas in consecutive time coordinates:

1. A cylinder shaped cluster was simulated with radius of the circular base and height equal to 0.198 and [3,6], respectively.

2. A cone shaped cluster was simulated as a frustum of a cone. The radius of lower and upper circular base were equal to 0.115 and 0.265, respectively. The time window was equal to [3,6].

3. An "L-3D"-shaped cluster was simulated with zone *L *= *L*_1 _∪ *L*_2 _where *L*_1 _= [0.3, 0.7] × [0.3, 0.7] × [3, 4], *L*_2 _= [0.484, 0.7] × [0.3, 0.7] × [5,6].

Given a cluster model, exactly the same sets of data were used for all algorithms. 10, 000 Monte Carlo simulations of the null hypothesis were performed, and also 10, 000 Monte Carlo replications for each one of the three alternative hypothesis models. The three measures above, namely, detection power, sensitivity an PPV were computed for the most likely cluster in each replication.

Table 2 presents the resulting average power, sensitivity and PPV for 10, 000 replications of each one of the three cluster models obtained with the VBScan and Elliptic PST algorithms. For all three space-time clusters, the power of detection of the VBScan was higher than the power of the Elliptic PST. This also occurs for PPV and Sensitivity. The results found in the three measures evaluated for "L-3D"-sh aped cluster show the greater flexibility of VBScan, compared with Elliptic PST method.

**Table 2:** Power, sensitivity and positive predicted value comparisons for the three alternatives space-time clusters.

Shaped cluster	Power		Sensitivity		PPV	
	
	Elliptic PST	VBScan	Elliptic PST	VBScan	Elliptic PST	VBScan
Cylinder	0.4789	0.6510	0.5447	0.6532	0.6415	0.6738
Cone	0.3863	0.5093	0.4683	0.5947	0.5822	0.6157
"L-3D"	0.3316	0.5768	0.4530	0.6141	0.5323	0.5943

### Dengue Fever Clusters

We describe an application to cases of dengue fever in the municipality of Lassance in southeast Brazil. Dengue fever is caused by one of four types of virus, typically transmitted by the mosquito *Aedes aegypti*. Immunity to one strain does not confer lifelong immunity to the other strains. Underreporting is a serious problem with dengue fever data. It is estimated that only 10% of the cases are usually registered at hospitals or health care units [33]. A pilot project was set in order to obtain more reliable data, with surveillance done at the individual level. Community health agents of the Family Health Program (FHP) [34] performed weekly visits at all residences within the municipality. This already existing program provides guidance for citizens and informs local public health authorities about possible health problems, and is highly regarded in the community. Due to its unique features, the FHP could in principle provide a huge amount of information which would be useful in the surveillance of many diseases, but data almost never is organized beyond local level. In our pilot project, data collected by 13 community health agents in the urban zone of the municipality of Lassance were compiled by two nurses, and sent for analysis every workweek with the assistance of the Secretary of Health and Epidemiological Surveillance in Lassance. In addition, home location was registered for every resident in the urban part of the city. In the period of six months in 2010, between January 12*th *and June 14*th*, a total of 57 cases were reported from a total of 3986 individuals in the population at risk.

**Figure 5:**
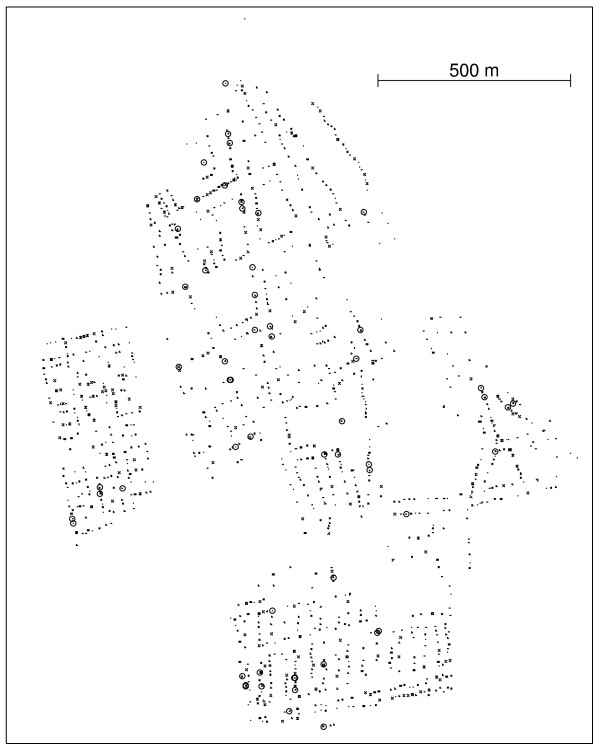
**Spatial distribution of the observed cases of dengue fever (circles) and controls (dots) in Lassance City, southeast Brazil**. North is up in the map.

**Figure 6:**
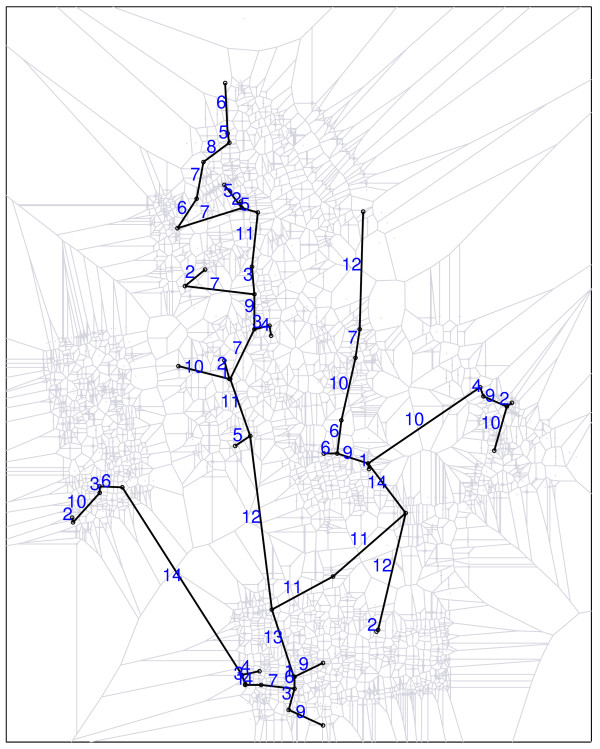
**Lassance City dengue fever map with assigned weight values for the edges of the Voronoi minimum spanning tree, along with the drawing of the Voronoi cells in the background (in gray)**.

The spatial distribution of the observed cases of dengue fever and controls in Lassance City is shown in Figure 5. We have included in Figure 6 the *δ*(*i*, *j*) values for the edges of the Voronoi minimum spanning tree along with the drawing of the Voronoi cells in the background (in gray).

Dengue is not transmitted directly from one person to another. The virus is transmitted to the mosquito *A. aegypti *after biting an infected individual. The mosquito can carry the virus for 10 to 14 days. In humans, the virus remains in an incubation period that may last from 3 to 15 days. Only after this period the symptoms can be observed. In this way, the study period was divided into 11 intervals of 14 days, as shown in Table 3.

**Table 3:** Study time period subdivided.

Time	days observed	cases
1	01-12 to 01-25	03
2	01-26 to 02-08	06
3	02-09 to 02-22	02
4	02-23 to 03-08	07
5	03-09 to 03-22	05
6	03-23 to 04-05	09
7	04-06 to 04-19	04
8	04-20 to 05-03	09
9	05-04 to 05-17	09
10	05-18 to 05-31	02
11	06-01 to 06-14	01

Each unit represents a period of 14 days.

#### Spatial analysis

We relied upon ordinary topographic maps and aerial images provided by Lassance's City Hall, because high resolution Google Earth images were not available [35].Those aerial images were manually matched with the existing topographic maps. Data are plotted in the map according to the exact location of each individual of the population at risk. Data are available as additional files. 1 &2. To detect possible clusters, the VBScan method was applied.

**Figure 7:**
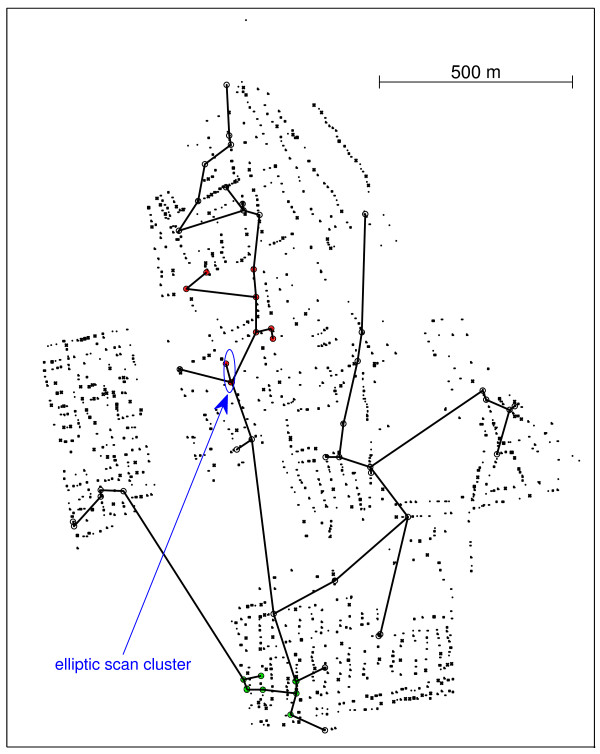
**Purely spatial primary (red dots) and secondary (green dots) dengue fever clusters found by the VBScan, and the primary cluster found by the Elliptic Scan**.

The two most likely clusters presented 10 and 9 cases, respectively for the primary and secondary clusters, as shown in Figure 7. For the primary cluster a p-value = 0.004 was found, see Table 4. Table 4 shows that the secondary cluster is also statistically significant. Those p-values a re computed from 999 Monte Carlo simulations under the null hypothesis. Hence, we conclude that there is evidence of a geographically significant high risk of dengue fever in some specific regions within the urban area of Lassance City.

**Table 4:** Match values for spatial clusters Dengue fever data set by using VBScan method

Clusters	LLR	cases	p-value
**primary**	17.5686	10	0.004
**secondary**	15.2390	09	0.016

Employing the elliptic scan, also with 999 Monte Carlo simulations, the most likely cluster found has only 3 cases, contained within the primary cluster found by VBScan, as marked in Figure 7 (p-value = 0.054). The run time for 999 Monte Carlo replications for the Dengue fever cluster was about 187 seconds for the VBScan and 764 seconds for the elliptic scan. This interesting result arises due to the peculiar features of this problem:

• The population does not follow a random-like spatial distribution; instead, the individuals are roughly aligned according the housing geometry of the streets.

• The neighborhood structure induced by the Euclidean metric, which is used by elliptic scan, becomes very different from the neighborhood structure induced by the Voronoi distance.

Specifically, the population densities, which are considered in the computation of both the scan statistics, are distinct, because the Voronoi distance is calculated along the edges that link the case points, while the density in the elliptic scan considers all individuals inside the ellipses. Clearly, this pattern of population spread causes the elliptic scan to consider a greater number of non-infected control cases inside a potential cluster than the VBScan, reducing the power of the Elliptic Scan. It can be noticed, in the primary cluster found by VBScan, that a path used by this algorithm to link a set of cases may avoid the directions in which a large number of non-infected individuals are located. This is due to the definition of Voronoi distance, which exactly assigns larger distances to such paths. The clusters, therefore, may include larger edges (in terms of Euclidean metric) which cross less crowded regions - these are the smaller edges in Voronoi distance - causing the opposite effect in the VBScan detection power.

The primary cluster (indicated by red points in Figure 7) has two edges crossing city blocks diagonally, both with assigned value *δ*(*i*, *j*) = 7, as can be seen in Figure 6. The longest (in terms of Euclidean distance) edge that links the two northwestern cases crosses a moderately high populated region, as measured by the Voronoi distance, is not an artifact. Although the interior part of the block crossed by this edge has no control individuals, there are many individuals living in its borders, implying that there are several Voronoi cells (bounded by gray lines in the background) inside the block, which in turn makes the diagonally crossing edge intercept several cells in its path. This is a fine example of how the Voronoi distance measures adequately the population density, as a composition of the individual cells (regions of influence) intercepted by the edge's path.

#### Detecting space-time clusters

The prospective space-time geographical surveillance system proposed here was applied for the detection of dengue fever space-time clusters over the same data set. The time window has a range of [1,11], in which each unit represents a period of 14 days, as set out in Table 3. The results are given in Table 5, whose first column indicates the temporal restriction for the construction phase of the minimum spanning tree, influencing the significance of the cluster detection.

**Table 5:** Match values for space-time clusters Dengue fever data set analysing the periods 1-11, by using VBScan method.

temporal length edge *τ*	cases	onset time of the disease for the cases	LLR	p-value
1	06	{7,8}	17.3207	0.003
2	07	{5,7,8}	15.0091	0.008
4	06	{7,8}	15.3053	0.019
6	10	{1,2,4,6,8,9}	15.7764	0.024
8	10	{1,2,4,6,8,9}	15.7764	0.024

**Figure 8:**
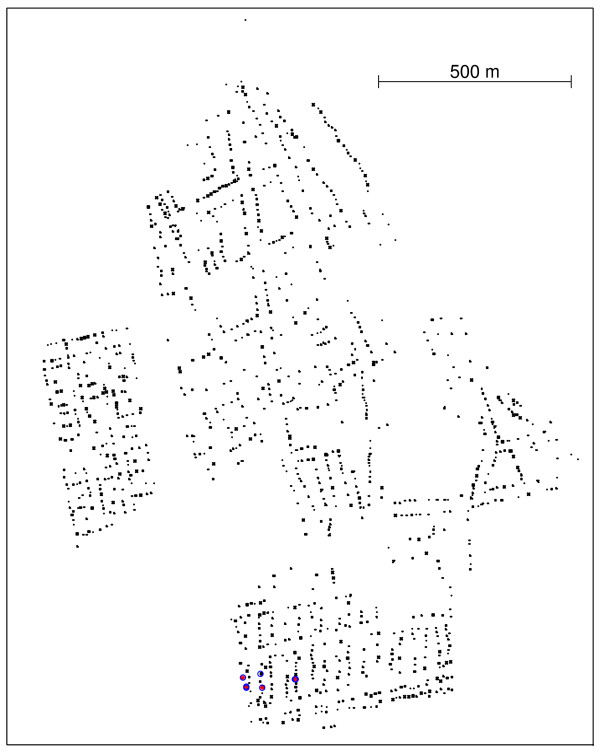
**Space-time clusters of the dengue fever dataset, with temporal constraint parameter values τ = 1(red stars) and τ = 2(blue circles), matching the values shown in lines 1 and 2 of Table 5, respectively **.

Table 5 shows that all clusters that were found are statistically significant for the time period [01-12 to 06-14]. Again, 999 Monte Carlo simulations were generated under null hypothesis. The two space-time clusters with smaller p-values are part of the secondary spatial cluster, as shown in Figure 8 and the values indicated by lines 1 and 2 respectively in Table 5.

The cluster that was found as the primary cluster in the purely spatial analysis does not appear as a cluster in the space-time analysis. In the first situation, the cases were spread along the time axis. On the other hand, only a few cases were included in the same cluster, when time is considered. This pattern suggests that, instead of a single space-time cluster of dengue fever, there was a series of several independent re-infections of individuals within the space region of that cluster. This interpretation is consistent with an environmental information: that region belongs to the central part of the municipality, where several public service facilities are located. This means that such a region receives a flow of people from all other regions, which is consistent with the hypothesis of several re-incidences of dengue fever cases in that region in events which are not directly dependent.

On the other hand, the cluster that was found as the secondary cluster in the purely spatial analysis appears as the single detected cluster in the space-time analysis. In this cluster, most of the cases occurred within a small temporal window. Located in a poorer part of the municipality, at the border of the urban area, this region has several environmental factors favoring a large concentration of mosquito larvae, such as deficient sewage installations and garbage collection, accumulated water puddles, and the presence of many vacant lots and houses. Furthermore, the timing of the cluster coincides with the rainiest weeks of 2010. These data are consistent with the hypothesis of a single event epidemics outbreak, with a direct causal correlation between the several cases.

## Conclusions

We developed and tested a novel algorithm for the detection and inference of space-time clusters for data sets, the Voronoi Based Space-Time Scan (VBScan). The concept of Minimum Spanning Tree (MST) is adapted with the novel Voronoi distance, which is used to compute the set of potential clusters. This set is then evaluated using the spatial scan statistic, producing the most likely cluster of cases.

The class of problems considered here assumes a point data set to represent the location of individuals in a population, classified either as controls or disease cases, within a limited domain in space-time. The cluster is modeled in space coordinates as a connected graph with tree structure, joining a subset of the disease cases, and in space-time coordinates as a sequence of such trees with space projections that have non-null intersection. A distance measure, named Voronoi distance, is proposed here in order to define a meaningful distance for the construction of a minimum spanning tree (MST) that represents the more likely connections between individuals, in a given graph. This structure allows the direct application of the scan statistics, with the calculation of the likelihood ratio of the estimated cluster.

The Voronoi distance between any two points may also be interpreted as an approximation to the line integral of the population density function over the segment joining those two points. For this reason, the Voronoi MST is the natural extension of the Euclidean MST, taking into account the heterogeneity of the population density. On the other hand, the Euclidean distance is an approximation to the corresponding line integral only when the map is cartogram transformed, in such a way that the population density becomes homogeneous. The Voronoi distance concept is employed once again in our method, after the collection of potential clusters is extracted from the Voronoi MST: it is used to estimate the number of control individuals under the region of influence of each one of the case individuals. This allows the definition of the population associated to each potential cluster, which may be evaluated through the spatial scan statistic.

By proposition 1, we attached a ball of radius *ω_i_*/2 to each case *c_i _*belonging to the cluster *S*. The value *ω_i _*was chosen as the minimum weight of the edges that are incident to *c_i _*in the Voronoi MST. An alternative definition may use the average (or even the median) of the weights of the edges that are incident to *c_i_*, instead of the minimum value of the weights. We have conducted numerical simulations suggesting that there are negligible differences of performance using these alternative definitions, compared with the original definition using the minimum value of the weights. This is a good indication that proposed definition of local population of the cluster is stable.

The results of numerical simulations show that the proposed algorithm, space-time VBScan, has higher power of detection, positive predictive value, sensitivity and computational speed than the space-time Elliptic Scan. The flexibility verified of VBScan allows an enhanced ability to deal with the variation of the disease spread along the time dimension.

An application was presented for Dengue fever incidence, with data available at individual level, in the municipality of Lassance, Brazil. Because we make use of an already existing team of community health agents, originally employed for health monitoring in general, Dengue fever surveillance is very cost effective in our setting, and we can focus our effort on mapping, data collection, data integrity issues and analysis. In a future work, we will use additional zoonosis and environmental data, and apply covariate analysis. This will allow better monitoring and forecasting of outbreaks.

VBScan also includes topological information from the point neighborhood structure, in addition to the usual geometric information. For this reason, it is more robust than purely geometric methods such as the elliptic scan. Those advantages were illustrated in a real setting for dengue fever space-time clusters, where the population spreads along a grid of straight lines according to the street mapping. It is worthy to notice that this kind of geometry of population distribution appears very often in urban environments. In those cases, the employment of VBScan should be recommended.

In the examples that we have analyzed, we observed that the Voronoi distance is very reliable to approximate the population heterogeneity, even for some unusual population distribution patterns, like a city block with zero individuals living in its interior and many individuals living on its borders.

One potential limitation of our analysis is the spatial mobility of individuals from their residences to workplace, which could impair the geographic delineation of the detected clusters. In a future work we will address this issue, using tools such as the workflow scan statistic [2].

The ability for the early detection of space-time clusters of disease outbreaks, when the number of points in the dataset is large, was shown to be feasible, due to the reduced computational load of the proposed methodology compared with classical methods. The proposed methodology is shown to present an enhanced power for the detection of space-time disease clusters.

## Competing interests

The authors declare that they have no competing interests.

## Authors' contributions

All the authors contributed to the methodology used in the study, wrote the necessary computer programs, conducted the simulations and data analysis, and drafted the manuscript. FCOM also coordinated the dengue fever data collection and the mapping of georeferenced individuals in Lassance City. All authors have read and approved the final manuscript.

## Additional Files

Data files of Dengue fever cases and controls in the urban region of Lassance city, Minas Gerais state, Brazil, for the time period between January 12th and June 14th 2010 are supplied.

## Supplementary Material

Additional file 1 **Controls coordinates**. Click here for file

Additional file 2 **Dengue fever cases coordinates and onset-date**. Click here for file
